# Assessment of brain cholesterol metabolism biomarker 24S-hydroxycholesterol in schizophrenia

**DOI:** 10.1038/s41537-020-00121-4

**Published:** 2020-11-20

**Authors:** Joshua Chiappelli, Maria S. Quinton, Dmitri Volfson, Michael Cwik, Wyatt Marshall, Heather Bruce, Eric Goldwaser, Mark Kvarta, Ann Summerfelt, Peter Kochunov, Patricio O’Donnell, Liyi Elliot Hong

**Affiliations:** 1 Maryland Psychiatric Research Center, Department of Psychiatry, University of Maryland School of Medicine, Baltimore, MD USA; 2 Takeda Pharmaceuticals, Neuroscience Translational Medicine, Cambridge, MA USA; 3 Takeda Pharmaceuticals, Quantitative Sciences, Cambridge, MA USA; 4 Takeda Pharmaceuticals, Clinical Biomarker Development and Innovation, Cambridge, MA USA

**Keywords:** Schizophrenia, Biomarkers

## Abstract

Plasma 24S-hydroxycholesterol mostly originates in brain tissue and likely reflects the turnover of cholesterol in the central nervous system. As cholesterol is disproportionally enriched in many key brain structures, 24S-hydroxycholesterol is a promising biomarker for psychiatric and neurologic disorders that impact brain structure. We hypothesized that, as schizophrenia patients have widely reported gray and white matter deficits, they would have abnormal levels of plasma 24S-hydroxycholesterol, and that plasma levels of 24S-hydroxycholesterol would be associated with brain structural and functional biomarkers for schizophrenia. Plasma levels of 24S-hydroxycholesterol were measured in 226 individuals with schizophrenia and 204 healthy controls. The results showed that levels of 24S-hydroxycholesterol were not significantly different between patients and controls. Age was significantly and negatively correlated with 24S-hydroxycholesterol in both groups, and in both groups, females had significantly higher levels of 24S-hydroxycholesterol compared to males. Levels of 24S-hydroxycholesterol were not related to average fractional anisotropy of white matter or cortical thickness, or to cognitive deficits in schizophrenia. Based on these results from a large sample and using multiple brain biomarkers, we conclude there is little to no value of plasma 24S-hydroxycholesterol as a brain metabolite biomarker for schizophrenia.

## Introduction

The brain has the highest level of cholesterol among all organs of the human body and contains about a quarter of the body’s cholesterol despite the fact that it has only about 2% of the total body weight. The brain contains such a disproportionate amount of the total cholesterol in large part due to the key role of cholesterol in myelin sheath and neuronal cell membranes^1^. Given the evidence of constant turnover of cholesterol in the brain, mechanisms must be in place for the replacement of cholesterol and maintenance of steady-state levels of sterols^2^. In the brain, a key mechanism for the excretion of cholesterol maybe conversion to the oxysterol 24S-hydroxycholesterol (24-OHC), which enters circulation from the brain to be further metabolized by the liver at a rate roughly equivalent to de novo synthesis of cholesterol within the brain^3^. The enzyme responsible for the synthesis of 24-OHC, cholesterol 24-hydroxylase, is located primarily within neurons of the hippocampus, cerebellum, and cerebral cortex^4^. Knockout or inhibition of CYP46A1, the gene for this enzyme, causes learning deficits, increased neuronal death, and increased levels of beta-amyloid peptides in mice^5,6^. 24-OHC also acts as a positive allosteric modulator of NMDA receptors^7^. Almost all of the 24-OHC present in plasma is derived from cholesterol metabolism within the brain^8^, making this an appealing biomarker to potentially monitor cholesterol turnover in the brain.

Previous studies have found increased plasma levels of 24-OHC in patients with Alzheimer’s disease^9,10^, though other studies have found decreased levels^11,12^; in part, these mixed findings could be due to evidence that 24-OHC is elevated in early or mild Alzheimer’s but decreased in more advanced illness^13,14^. Plasma levels of 24-OHC are also decreased in Huntington’s disease^15,16^. These findings have led to efforts to identify pharmacological agents that target CYP46A1 as potential therapeutic agents for neurodegenerative disorders^17,18^.

In schizophrenia, structural brain abnormalities have been widely reported^19^. Furthermore, indices of brain structures such as white matter microstructure and other brain measures may exhibit a decline following illness onset that may be greater than typical age-related decline^20,21^, indicating a possible neurodegenerative component to the illness. Schizophrenia has been associated with lipid abnormalities, including abnormalities in plasma cholesterol levels evident even in the early phase of illness and in patients with little to no exposure to antipsychotic medications^22^. We thus hypothesized that brain structural changes may be associated with greater cholesterol breakdown or turnover, leading to higher 24-OHC levels in schizophrenia patients compared with controls. To our knowledge, no previous study has reported on plasma levels of 24-OHC in schizophrenia.

We also hypothesized that 24-OHC could represent a convenient peripheral proxy measure for brain structural and cognitive abnormalities in patients with schizophrenia. We chose two structural brain measures and two key cognitive measures to evaluate the potential role of 24-OHC. A highly replicable structural brain abnormality closely related to cognitive deficits in schizophrenia is abnormal white matter microstructure, as measured with diffusion tensor imaging^23,24^. Given that cholesterol is a major component of myelin, we hypothesized that fractional anisotropy (FA) of white matter tracts would be correlated with peripheral 24-OHC, since this molecule reflects cholesterol turnover in the brain. Cortical thickness is a measure of gray matter that is sensitive to atrophy occurring early in the course of Alzheimer’s disease^25^ and is also decreased in schizophrenia^19^. As some previous studies have found levels of 24-OHC to be related to measures of gray matter volume^12,26^, we hypothesized plasma 24-OHC levels would be correlated with cortical thickness. Finally, some of the most robust cognitive deficits in schizophrenia are working memory and processing speed deficits that may reflect the underlying gray and white matter structural abnormalities in schizophrenia^24,27^. Therefore, we also examined whether 24-OHC would be associated with these cognitive measures.

## Results

### Group differences

**Fig. 1:**
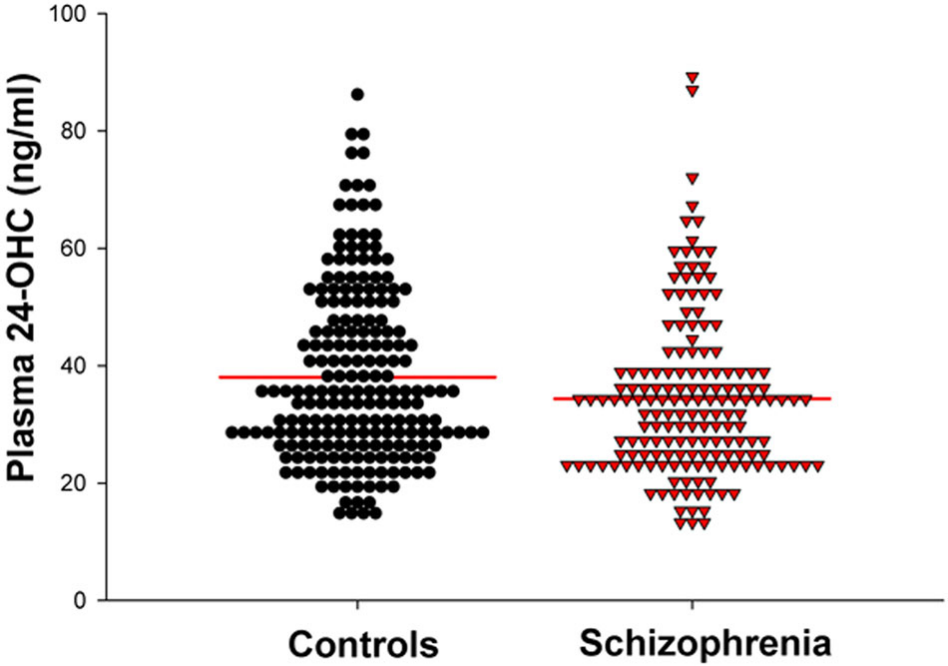
Dot density plot comparing plasma levels of 24S-hydroxycholesterol (24-OHC) in controls and individuals with schizophrenia spectrum disorder. Horizontal red lines indicate group means.

Patients (M = 35.8 ± 14.2 ng/ml) and controls (M = 38.0 ± 14.8 ng/ml) did not have significantly different levels of 24-OHC (F(1,425) = 0.07, *p* = 0.79; Fig. 1). There was a significant sex effect (F(1,425) = 17.91, *p* < 0.001) with females having higher levels (M = 40.3 ± 15.2 ng/ml) compared to males (M = 34.5 ± 13.6 ng/ml), but no significant sex × diagnosis effect. The sex effect was significant in patients (F(1,223) = 17.1, *p* < 0.001) and a similar trend was observed in controls (F(1,201) = 3.46, *p* = 0.064). When body surface area is included as a covariate in this model, the sex effect is no longer significant (*p* = 0.08), and instead, body surface area is significantly related to 24-OHC levels (F = 9.67, *p* = 0.002). In the subset of participants for whom total cholesterol levels were available, there were no significant effects of diagnosis ((F1,104) = 0.80, *p* = 0.37), sex ((F1,104) = 1.10, *p* = 0.30), or diagnosis × sex interaction ((F1,104) = 1.55, *p* = 0.22) on 24-OHC/total cholesterol ratio. Total cholesterol and 24-OHC were significantly correlated (*r* = 0.23, *p* = 0.017).

**Fig. 2:**
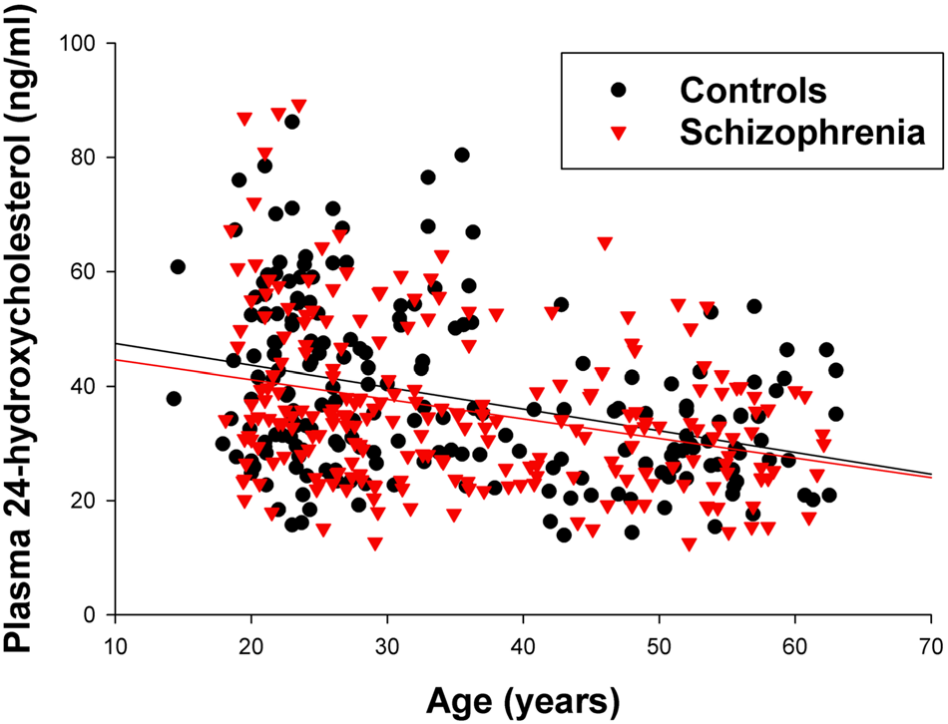
Scatterplot of relationship between age and plasma levels of 24S-hydroxycholesterol.

Age was significantly and negatively correlated with levels of 24-OHC in both controls (*r* = −0.35, *p* < 0.001) and patients (*r* = −0.31, *p* < 0.001) (Fig. 2), thus the age effect on 24-OHC was replicable across groups.

### Relationship of 24-OHC to white matter structure

In the sub-sample of individuals with DTI data available (*n* = 250, including 140 patients and 110 controls), patients had significantly reduced whole-brain white matter averaged FA (F(1,246) = 29.3, *p* < 0.001). Plasma levels of 24-OHC were not significantly associated with whole-brain white matter averaged FA when accounting for age, sex, diagnosis, and current smoking status (β = −0.07, *p* = 0.27). In the subset of participants with both DTI and total cholesterol data available (*n* = 69), 24-OHC/total cholesterol ratio was not associated with average FA (β = −0.09, *p* = 0.51).

### Relationship of 24-OHC to cortical thickness

In the subset of participants with structural imaging data available, individuals with schizophrenia had significantly lower cortical thickness compared to controls (F(1,122) = 9.75, *p* = 0.002, with age and sex as covariates). While cortical thickness and plasma 24-OHC are positively correlated when confounding variables are not accounted for (*r* = 0.259, *p* = 0.003), plasma 24-OHC was not significantly associated with cortical thickness in linear regression with age, sex, and diagnosis as covariates (β = 0.07, *p* = 0.39).

### Cognition

In the entire sample, levels of 24-OHC were correlated with working memory performance (β = 0.10, *p* = 0.048). However, in examining each group independently this trend was only found for controls (β = 0.22, *p* = 0.007) and was not present in patients (β = -0.02, *p* = 0.81). 24-OHC was not significantly associated with processing speed in the entire sample or in either group examined independently.

### Influence of smoking and medications

Smoking was associated with lower levels of 24-OHC after controlling for age and sex (β = −0.11, *p* = 0.017). The use of lipid-lowering medication was not associated with 24-OHC (β = −0.06, *p* = 0.22). Chlorpromazine dose equivalent of antipsychotic medication was not associated with 24-OHC in patients (β = −0.12, *p* = 0.084). In order to more fully explore potential medication effects, we coded participants by whether they were taking particular classes of medications or not, including typical antipsychotics, atypical antipsychotics, mood stabilizers, anticholinergics, and antidepressants. In an omnibus linear regression, including age and sex as covariates, and including binary variables for use of the above medication classes, we did not find evidence that any class of medication was significantly associated with 24-OHC levels. Similarly, in separate regression analyses in which each medication class is examined alone, no significant effects were found.

## Discussion

This study found no evidence to indicate that plasma levels of 24-OHC are abnormal in people with schizophrenia compared to healthy controls. We found no evidence to support the hypothesis that 24-OHC could be a biomarker related to cognitive impairment or structural brain changes in schizophrenia. Given the substantial sample size for this study, the testing of multiple clinical and biological markers, and the consistent sex and age effects between groups, these negative findings lead us to conclude that there is little to no utility of plasma 24-OHC as a biomarker in schizophrenia research.

In our sample, prominent inverse relationships were observed between age and plasma 24-OHC in both patients and controls. We also observed the effects of sex on plasma 24-OHC, with women having higher levels than men; at least one prior study has found similar results^28^. These age and sex effects are likely explained by previous findings that plasma 24-OHC levels are influenced by the relative ratio of total brain volume to liver volume. CYP46A1 is primarily expressed in neurons within the brain^4^, though increased expression in astrocytes has been observed in patients with Alzheimer’s disease^29^. Plasma 24-OHC has been hypothesized to reflect the mass of metabolically active neurons^30^, and consistent with this hypothesis several studies have found positive relationships between plasma 24-OHC and volume of the hippocampus, total brain volume, or gray matter volume^12,26,31^. In our sample, there was a positive association between 24-OHC and cortical thickness, but this association was highly attenuated when controlling for age. Greater liver volume may be associated with greater metabolism of circulating 24-OHC to bile acids^11^. As liver volume is proportional to body surface area^32^ the smaller body size of women may explain the sex difference in plasma 24-OHC levels. Consistent with this, when the body surface area is included as a covariates in our analyses, the effect of sex on plasma 24-OHC is highly attenuated. Similarly, an early study of plasma 24-OHC found that levels are very high in childhood, when the ratio of total brain volume to liver volume is relatively high^11^, but found no difference between early and late adulthood^8^. Another study of primarily older adults found no correlation of plasma 24-OHC with age^14^, but a larger study with a wide age range of participants found a modest negative correlation between age and 24-OHC levels^31^. In rodent studies, brain 24-OHC levels developmentally follow a bell-shaped pattern with low levels during early postnatal stages, greatly elevated levels with maturation into young adulthood, and levels declining with aging^8,33^. Data from the current study is more consistent with rodent findings and the large sample study of Stiles and colleagues^31^; the larger sample size and wide age range of participants in this study may have allowed detection of this age-related trend, which may be secondary to the gradual decrease in gray matter volume with aging.

As 24-OHC represents one of the few ways for the CNS to remove excess cholesterol, we had hypothesized that its plasma levels could reflect the amount of myelin remodeling processes, and therefore might be related to white matter microstructure. This was not the case for either controls or individuals with schizophrenia. However, this does not rule out a possible association of plasma 24-OHC and measures of brain structure in conditions with more aggressive neurodegenerative disorders such as Alzheimer’s disease. Our interest in 24-OHC as a biomarker in schizophrenia was also spurred by findings that 24-OHC has allosteric effects on NMDA receptors^34^, as NMDA receptor antagonists such as ketamine can induce psychotic symptoms, and NMDAR hypofunction is associated with working memory and other cognitive deficits in schizophrenia^35,36^. However, our data only showed a nominal trend for the association of higher 24-OHC with better working memory in controls. This may be consistent with the NMDAR allosteric effect of 24-OHC, although the effect appeared small.

We found little evidence of medication effects on circulating levels of 24-OHC. There have been several clinical trials of HMG-CoA reductase inhibitors that examined their effects on 24-OHC levels; several but not all of these studies found that statins decreased plasma 24-OHC^37–39^. In our data, participants taking cholesterol-lowering medications had lower levels of 24-OHC, but this finding was not statistically significant. This may be due to the cross-sectional nature of this study, and the overall small number of participants using these types of medications. Antipsychotics are well-known to have effects on cholesterol levels, though it is unclear if these medications impact cholesterol metabolism within the CNS. We did not find evidence that antipsychotics or other classes of psychotropic medications had an effect on 24-OHC. However, this lack of medication effect should be interpreted cautiously, again due to the cross-sectional nature of this study, and the lack of data about medication adherence or past exposure to other medications.

This study is limited by the lack of measurement of 24-OHC in the brains of schizophrenia patients, leaving open the question if brain sterol metabolism is abnormal despite apparently normal 24-OHC levels in plasma. We did not measure cholesterol synthesis precursors such as lanosterol, lathosterol, and desmosterol; a more comprehensive lipidomic analysis may be necessary to determine if there are changes in CNS cholesterol metabolism in schizophrenia. Furthermore, total cholesterol levels were only available for a subset of participants, though the available data did not indicate that using the ratio of 24-OHC to total cholesterol would have produced different findings regarding brain structure or cognitive measures. Our clinical sample included both patients in the early course of the illness as well as patients with chronic illness, who were mostly clinically stable at the time of the study. We thus cannot rule out the possibility that plasma 24-OHC levels could be abnormal in a particular sub-type of psychotic illness, though the overall negative results of this study suggest this possibility is unlikely.

Circulating levels of 24-OHC represent a promising biomarker for neurodegenerative disorders, and recent work suggests the clinical potential of targeting the production of this metabolite in neurodegenerative diseases^17,18,40,41^. Our results are consistent with the hypothesis that plasma 24-OHC levels reflect a balance between the amount of metabolically active neurons that produce 24-OHC and hepatic metabolism, based on the sex differences on 24-OHC levels. However, our results suggest plasma levels of 24-OHC are likely normal in schizophrenia and cannot explain key brain structural or cognitive abnormalities observed in the illness.

## Methods

### Participants

The patient sample included 226 individuals with schizophrenia spectrum disorder (174 with schizophrenia, 48 with schizoaffective disorder, and 4 with schizophreniform or unspecified psychotic disorder), were recruited from local outpatient mental health clinics. Control participants (*n* = 204) were recruited using media advertisements. The Structured Clinical Interview (SCID) for DSM-IV was used to confirm diagnoses, and to determine that control participants had no current DSM-IV Axis I diagnoses. Participants were excluded from the study if they had a history of major neurological illnesses (including epilepsy, cerebrovascular accident, and head injury with cognitive sequelae) or any uncontrolled major medical illnesses. Participants were excluded if they had substance dependence within 6 months prior to the study, or current substance use disorder (except nicotine or cannabis). Medication information was unavailable for 17 patients. For those with complete medication information, 13 patients were not taking antipsychotics at the time of the study, 155 were taking atypical antipsychotics, 25 were taking typical antipsychotics, and 16 were taking a combination of antipsychotic types (Table 1). Of the patients on atypical, 40 were on clozapine. In addition, 39 patients were also on mood stabilizers, 82 were taking an antidepressant, and 42 were on anticholinergic medications. Nine control participants and 23 patients were taking a lipid-lowering medication at the time of the study. Participants gave written informed consent approved by the University of Maryland Baltimore IRB.

### Clinical assessments

To assess processing speed and working memory, participants were tested with the Digit Symbol Coding task of the WAIS-3 and the Digit Sequencing task from the Brief Assessment of Cognition in Schizophrenia, respectively^42,43^. Slower processing speed and reduced working memory capacity are among the most robust cognitive impairments in schizophrenia^44,45^. Height and weight were obtained by self-report, and body surface area was calculated according to the geometric formula^46^ (Table 1).

**Table 1:** Summary of demographic characteristics.

	Control (*n* = 204)	Schizophrenia (*n* = 226)	Test-statistic	*p*-value
Age (years)	34.7 ± 13.6	35.6 ± 12.9	t = −0.71	0.28
Smoker/Non-smoker	60/144	85/141	χ^2^ = 3.23	0.073
Male/Female	98/106	156/70	χ^2^ = 19.5	<0.001
Body surface area (m^2^)	1.96 ± 0.27	2.04 ± 0.29	t = 2.56	0.011
Race				
White	117	93		
Black	68	119	χ^2^ = 17.0	<0.001
Asian	15	9	χ^2^ = 3.08	0.079
Other	4	5		
Antipsychotic medication^a^				
Typical	—	25		
Atypical	—	155		
Combination of typical and atypical	—	16		
Unmedicated	—	13		
Antidepressant^a^	—	82		
Mood stabilizer^a^	—	39		
Anticholinergic medication^a^	—	42		
Lipid-lowering medication^a^	9	23	χ^2^ = 5.47	0.019

Variance reported as ± standard deviation.

^a^Current medication information was missing for 17 participants.

### Measurement of 24-OHC

Whole blood was collected in EDTA-containing tubes (Vacutainer) which were immediately centrifuged at 2500 rpm for 10 min. Plasma was then stored at −80 °C until assay. The concentrations of 24-OHC in human plasma samples were analyzed at CMIC, Inc. (Hoffman Estates, IL) by high-performance liquid chromatography/tandem mass spectrometry (LC–MS/MS). The analyte (24-OHC) and internal standards (24-(S/R) Hydroxycholesterol -d7, Avanti Polar Lipids, Inc.) were extracted from human plasma (500 μL) by a liquid–liquid extraction procedure and detected by multiple reaction monitoring. Using 24-(S) Hydroxycholesterol -d7 as the internal standard (IS), 24S-Hydroxycholesterol concentrations were calculated using Analyst software 1.5.2 with linear regression using the least-squares method (with 1/x2 weighting) with a lower limit of quantification (LLOQ) of 2 ng/mL and upper limit of quantification of 100 ng/mL. The validity of this method was supported with preliminary tests of selectivity, matrix effects, intra- and inter-assay precision and accuracy, recovery, stability in plasma (long-term freezer storage, exposure to freeze/thaw cycle), post-preparative stability (re-injection reproducibility, extract stability and batch length stability), stock and working solution stability, dilution integrity, lower limit of quantitation, carryover and cross analyte interference.

To assess whether total plasma cholesterol may bias the 24-OHC assessment, available total cholesterol in a subset of participants who had completed another study around the same time as completion of assessments for the current study^47^ was used for association analysis and ratio calculation with 24-OHC. Fasting blood samples were sent to a Clinical Laboratory Improvement Amendments (CLIA)-certified commercial laboratory for analysis of total cholesterol.

### Neuroimaging

All imaging was performed at the University of Maryland Center for Brain Imaging Research using a Siemens 3 T TRIO MRI (Erlangen, Germany) system equipped with a 32-channel phase array head coil.

Diffusion Tensor Imaging (DTI): A subgroup of participants completed the high-angular resolution diffusion imaging (HARDI) DTI collected using a single-shot, echo-planar, single refocusing spin-echo, T2-weighted sequence with a spatial resolution of 1.7 × 1.7 × 3.0 mm and the following sequence parameters: TE/TR = 87/8000 ms, FOV = 200 mm, axial slice orientation with 50 slices and no gaps, five *b* = 0 images and 64 isotropically distributed diffusion-weighted directions with *b* = 700 s/mm^2^. These parameters were chosen to maximize the contrast to noise ratio for FA measurements^48^. FA images were created by fitting the diffusion tensor to the motion and eddy current diffusion data. RMSDIFF^49^ was used to estimate the distance between diffusion sensitized and *b* = 0 images. All data passed quality assessment control of <3 mm accumulated motion during the scan. FA images were then spatially normalized to the Johns Hopkins University atlas^50^ and then nonlinearly aligned to a group-wise, minimal-deformation target (MDT) brain using the FLIRT method^49,51^. Next, individual FA images were averaged to produce a group-average skeleton of white matter tracts. Finally, images were thresholded at FA = 0.20 level to eliminate non-white matter voxels, and FA values were projected onto the group-wise skeleton of white matter structures, to account for residual misalignment among individual tracts. FA values were assigned to each point along a skeleton using the peak value found within a designated range perpendicular to the skeleton. For the current study, only whole-brain tract-averaged FA was used for statistical analysis. The DTI methods and data reported here have been previously published^24,52^.

Cortical thickness: A small subset of participants (*n* = 60 patients and 66 controls) completed high-resolution, T1-weighted structural imaging using 3D Turbo-flash sequence with an adiabatic inversion contrast pulse with the following scan parameters: TR/TI/TE = 2100/785/3.04 ms, flip angle = 13°, voxel size (isotropic)=0.8 mm. To improve signal-to-noise ratio and reduce motion artifacts, each participant was scanned 5 times consecutively using the same protocol, and these images were then linearly coregistered and averaged^53^. The analysis followed the procedures described by Fischl and Dale^54^. Images were corrected for magnetic field inhomogeneities, affine-registered to the Talairach–Tournoux atlas and skull-stripped. White matter voxels were identified based on the location and intensity and were grouped into a mass of connected voxels using a six-neighbor connectivity scheme. A mesh of triangular faces was built around the white matter, using two triangles per exposed voxel face. The mesh was smoothed using trilinear interpolation. Topological defects were corrected to ensure that the surface had the same topological properties of a sphere^54^. The second iteration of smoothing was applied to yield a realistic representation of the interface between gray and white matter. The external cortical surface, corresponding to the gray matter, was produced by expanding the white matter surface outwards while maintaining constraints on its smoothness and on the possibility of self-intersection^54^. The white matter and gray matter surfaces were parcellated into smaller regions using an automated process^55^. This was done by first homeomorphically mapping the pial surface to a spherical coordinate system, where the folding patterns were matched to an average map^54^. An a priori atlas of probabilities for regions of interest was used in a Bayesian approach to establish probabilities that a given vertex belongs to a certain label. In a second, iterative step, the surface was treated as an anisotropic, non-stationary Markov random field, where for each vertex, the labels assigned to its neighbors were considered. The labeling was iterated until no vertices changed their assignments^54^. Cortical thickness was calculated by measuring the distance between gray matter and white matter polygonal meshes. The whole-brain gray matter thickness measurement was obtained by averaging gray matter thickness of individual cortical areas across the left and right hemispheres. These methods and most of the cortical thickness data reported in this manuscript have also been published previously^56^.

### Statistical analyses

The primary analyses were: (1) an ANCOVA examining differences in 24-OHC with diagnosis as independent factors and age and sex as covariates, and (2) separate linear regression analyses to determine if 24-OHC levels were associated with white matter tract-averaged FA, cortical thickness, processing speed, or working memory, with age, sex, and diagnosis as covariates. Secondary analyses explored if the ratio of 24-OHC/total cholesterol were different between patients and controls, or related to imaging and cognitive variables, and to explore medication and smoking effects on 24-OHC. As plasma levels of 24-OHC may be influenced by body surface area^11^, we also explored the influence of height as a covariate in the above analyses.

### Reporting summary

Further information on research design is available in the Nature Research Reporting Summary linked to this article.

## Supplementary information

Reporting Summary

## Data Availability

The dataset generated and analyzed in the current study is available from the corresponding author on reasonable request.
