# Supplementary material for: Assessment of brain cholesterol metabolism biomarker 24S-hydroxycholesterol in schizophrenia
Source: NPJ Schizophr. 2020 Nov 20;6:34. doi: 10.1038/s41537-020-00121-4 (PMC7680117; doi:10.1038/s41537-020-00121-4)
Supplement: Supplementary file 1 — Reporting Summary [file 41537_2020_121_MOESM1_ESM.pdf]

nature research

Corresponding author(s): Chiappelli, Joshua

Last updated by author(s): Jul 16, 2020

Reporting Summary

Nature Research wishes to improve the reproducibility of the work that we publish. This form provides structure for consistency and transparency in reporting. For further information on Nature Research policies, see our Editorial Policies and the Editorial Policy Checklist.

Statistics

For all statistical analyses, confirm that the following items are present in the figure legend, table legend, main text, or Methods section.

n/a	Confirmed	
☐	☒	The exact sample size (*n*) for each experimental group/condition, given as a discrete number and unit of measurement
☐	☒	A statement on whether measurements were taken from distinct samples or whether the same sample was measured repeatedly
☐	☒	The statistical test(s) used AND whether they are one- or two-sided<br>*Only common tests should be described solely by name; describe more complex techniques in the Methods section.*
☐	☒	A description of all covariates tested
☐	☒	A description of any assumptions or corrections, such as tests of normality and adjustment for multiple comparisons
☐	☒	A full description of the statistical parameters including central tendency (e.g. means) or other basic estimates (e.g. regression coefficient) AND variation (e.g. standard deviation) or associated estimates of uncertainty (e.g. confidence intervals)
☐	☒	For null hypothesis testing, the test statistic (e.g. *F*, *t*, *r*) with confidence intervals, effect sizes, degrees of freedom and *P* value noted<br>*Give P values as exact values whenever suitable.*
☒	☐	For Bayesian analysis, information on the choice of priors and Markov chain Monte Carlo settings
☒	☐	For hierarchical and complex designs, identification of the appropriate level for tests and full reporting of outcomes
☒	☐	Estimates of effect sizes (e.g. Cohen's *d*, Pearson's *r*), indicating how they were calculated

Our web collection on statistics for biologists contains articles on many of the points above.

Software and code

Policy information about availability of computer code

Data collection	Not applicable
Data analysis	SPSS 12.0 for Windows

For manuscripts utilizing custom algorithms or software that are central to the research but not yet described in published literature, software must be made available to editors and reviewers. We strongly encourage code deposition in a community repository (e.g. GitHub). See the Nature Research guidelines for submitting code & software for further information.

Data

Policy information about availability of data

All manuscripts must include a data availability statement. This statement should provide the following information, where applicable:

- Accession codes, unique identifiers, or web links for publicly available datasets
- A list of figures that have associated raw data
- A description of any restrictions on data availability

The dataset generated and analyzed in the current study is available from the corresponding author on reasonable request.

Field-specific reporting

Please select the one below that is the best fit for your research. If you are not sure, read the appropriate sections before making your selection.

☒ Life sciences ☐ Behavioural & social sciences ☐ Ecological, evolutionary & environmental sciences

For a reference copy of the document with all sections, see nature.com/documents/nr-reporting-summary-flat.pdf

Life sciences study design

All studies must disclose on these points even when the disclosure is negative.

Sample size	No sample size calculations performed; all available samples from participants who met inclusion criteria were included
Data exclusions	No data excluded from analysis
Replication	No replication performed; primary results were negative
Randomization	Case control design, no randomization
Blinding	Laboratory assessment of 24-OHC levels in plasma samples were performed blind to participant diagnosis

Reporting for specific materials, systems and methods

We require information from authors about some types of materials, experimental systems and methods used in many studies. Here, indicate whether each material, system or method listed is relevant to your study. If you are not sure if a list item applies to your research, read the appropriate section before selecting a response.

Materials & experimental systems

n/a	Involved in the study	
☒	☐	Antibodies
☒	☐	Eukaryotic cell lines
☒	☐	Palaeontology and archaeology
☒	☐	Animals and other organisms
☐	☒	Human research participants
☒	☐	Clinical data
☒	☐	Dual use research of concern

Methods

n/a	Involved in the study	
☒	☐	ChIP-seq
☒	☐	Flow cytometry
☐	☒	MRI-based neuroimaging

Human research participants

Policy information about studies involving human research participants

Population characteristics	Participants included individuals with psychotic disorder confirmed by structured clinical interview, or individuals with no current psychiatric diagnosis as determined by structured clinical interview. Age range was 14-63; individuals over age 63 were excluded from parent study. Both males and females included.
Recruitment	Patients were recruited from outpatient clinics including those of our research center specializing in psychotic disorders, as well as area community psychiatry clinics. Comparison participants were recruited through advertisements.
Ethics oversight	University of Maryland Baltimore IRB

Note that full information on the approval of the study protocol must also be provided in the manuscript.

Magnetic resonance imaging

Experimental design

Design type	Structural and DTI only
Design specifications	not applicable
Behavioral performance measures	not applicable

Acquisition

Imaging type(s): DTI, structural

Field strength: 3T

Sequence & imaging parameters: 3D Turbo-flash sequence with an adiabatic inversion contrast pulse with the following scan parameters: TR/TI/TE=2100/785/3.04 ms, flip angle=13°, voxel size (isotropic)=0.8 mm, repeated 5 times

Area of acquisition: whole brain scan

Diffusion MRI: ☒ Used ☐ Not used

Parameters: single-shot, echo-planar, single refocusing spin-echo, T2-weighted sequence with a spatial resolution of 1.7 x 1.7 x 3.0 mm. The sequence parameters were: TE/TR=87/8000ms, FOV=200mm, axial slice orientation with 50 slices and no gaps, five b=0 images and 64 isotropically distributed diffusion weighted directions with b= 700 s/mm2.

Preprocessing

Preprocessing software: FreeSurfer

Normalization: details in methods section of manuscript

Normalization template: Talairach–Tournoux atlas

Noise and artifact removal: not applicable

Volume censoring: not applicable

Statistical modeling & inference

Model type and settings: not applicable

Effect(s) tested: summary measures (cortical thickness and white matter tract-averaged FA) only used, in linear regression models

Specify type of analysis: ☒ Whole brain ☐ ROI-based ☐ Both

Statistic type for inference (See Eklund et al. 2016): not applicable

Correction: not applicable

Models & analysis

n/a	Involved in the study
☒	☐ Functional and/or effective connectivity
☒	☐ Graph analysis
☒	☐ Multivariate modeling or predictive analysis
